# Early nucleolar disorganization in *Dictyostelium* cell death

**DOI:** 10.1038/cddis.2016.444

**Published:** 2017-01-05

**Authors:** M F Luciani, Y Song, A Sahrane, A Kosta, P Golstein

**Affiliations:** 1 Centre d'Immunologie de Marseille-Luminy, Aix Marseille Université, Inserm, CNRS, Marseille, France; 2 Microscopy Core Facility, FR3479 Institut de Microbiologie de la Méditerranée, CNRS, Marseille, France

## Abstract

Cell death occurs in all eukaryotes, but it is still not known whether some core steps of the cell death process are conserved. We investigated this using the protist *Dictyostelium*. The dissection of events in *Dictyostelium* vacuolar developmental cell death was facilitated by the sequential requirement for two distinct exogenous signals. An initial exogenous signal (starvation and cAMP) recruited some cells into clumps. Only within these clumps did subsequent cell death events take place. Contrary to our expectations, already this initial signal provoked nucleolar disorganization and irreversible inhibition of rRNA and DNA synthesis, reflecting marked cell dysfunction. The initial signal also primed clumped cells to respond to a second exogenous signal (differentiation-inducing factor-1 or c-di-GMP), which led to vacuolization and synthesis of cellulose encasings. Thus, the latter prominent hallmarks of developmental cell death were induced separately from initial cell dysfunction. We propose that (1) in *Dictyostelium* vacuolization and cellulose encasings are late, organism-specific, hallmarks, and (2) on the basis of our observations in this protist and of similar previous observations in some cases of mammalian cell death, early inhibition of rRNA synthesis and nucleolar disorganization may be conserved in some eukaryotes to usher in developmental cell death.

Developmental cell death has been observed in most if not all multicellular eukaryotes where it has been looked for. This *ubiquity* of developmental cell death in multicellular eukaryotes argues in favor of conserved core mechanisms. Developmental cell death in different organisms can, however, be of distinct morphological types. This *polymorphism* may speak in favor of lineage-specific hallmarks, selected by evolution as a function of the organism and circumstances. How to reconcile possible conservation and polymorphism? Which mechanism may be conserved? A convenient model to study these questions is *Dictyostelium*, whose cell death shows prominent hallmarks such as vacuolization and cellulose encasing.

The protist *Dictyostelium discoideum* multiplies in rich medium as a unicellular organism. Starvation triggers aggregation and further morphogenesis, leading within 24 h to a 1–2 mm high mature fruiting body made of a mass of spores on top of a stalk. This stalk is made of dead or dying cells unable to re-grow in rich medium.^1^ Each of these stalk cells shows a very large vacuole and cellulose encasing.^2, 3^ The resulting vacuolar pressure and cellulose wall counterpressure mechanically reinforce the stalk, thus optimize spore dissemination. Vacuoles and cellulose walls are therefore considered to confer a selective advantage.

*Dictyostelium* cell death in stalks could be mimicked and more easily studied *in vitro* in monolayers.^4^ Two signals were required for full induction of this cell death. The initial signal starvation plus cAMP led to the appearance of autophagosomes and autophagolysosomes,^5, 6^ thus of signs of autophagy. Second signal exogenous differentiation-inducing factor-1 (DIF-1)^7^ led to polarized ‘paddle cells',^8^ which rounded up, acquired a cellulose encasing and a large vacuole that progressively occupied most of the cell volume.^8, 9^ The cyclic dinucleotide c-di-GMP was recently found to be able to act as a second signal *in vitro*.^10, 11^ Random insertional mutagenesis has helped identify some of the molecules involved in signaling by DIF-1 (refs 12, 13) but not by c-di-GMP.^11^ In cells where autophagy had been genetically inhibited, addition of DIF-1 led to a shift from vacuolar to necrotic cell death.^14, 15, 16^ Altogether, *Dictyostelium* cells in monolayers offer a model of non-apoptotic, non-necrotic, two-signal-induced cell death with vacuolization and cellulose encasing.^12^

We show here that upon initial signaling, cells in clumps were not only primed to respond to the second signal, but already showed severe dysfunction. This appeared as irreversible inhibition of rRNA and DNA synthesis and depletion of nucleolar rRNA stores, together with nucleolar disorganization and autophagy at the ultrastructural level, without, however, immediate loss of membrane integrity. Thus, the initial signal (starvation plus cAMP) led to both marked cell dysfunction and priming for the second signal, and the second signal (DIF-1 or c-di-GMP) induced hallmarks of death, namely vacuolization and cellulose encasing. These results may thus reflect a two-step process, a first step conserved in at least some instances of eukaryotic cell death, followed by a more organism-specific step, accounting for both ubiquity/conservation and polymorphism. Also, together with similar previous observations in some cases of mammalian cell death, these results suggest that initial signal-induced inhibition of rRNA synthesis and nucleolar disorganization may be conserved as early steps of developmental cell death throughout eukaryotes.

## Results

### An initial signal led to clumped cells primed to respond to second signals

**Figure 1:**
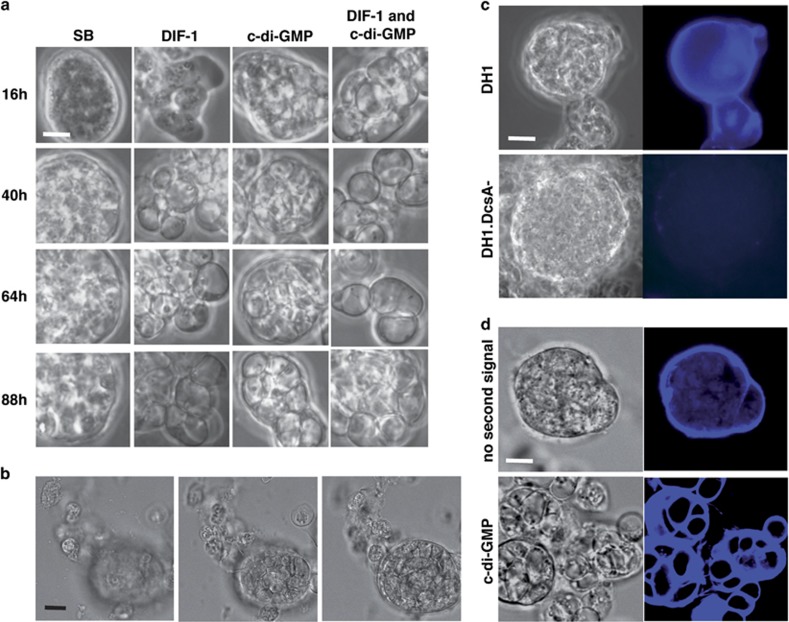
*Dictyostelium* cells in cAMP-induced clumps were primed for vacuolization and cellulose encasing upon second signaling. (**a**) Induction of cell death. DH1 cells starved for 8 h in SB saline in the presence of cAMP were further incubated for the indicated times, either in SB alone, or in SB containing the inducers DIF-1, c-di-GMP, or DIF-1 and c-di-GMP. SB and cAMP led to enveloped clumps, and addition of inducers led to vacuolization. Note the integrity of c-d-GMP-treated enveloped clumps within which vacuolization occurred. This pattern was found in each of close to 200 independent experiments including groups with or without c-di-GMP. From another point of view, each LabTek chamber seeded with 3 × 10^5^ cells showed about 100 clumps, each containing an average of 30 cells, thus only a minority of cells were recruited into clumps. (**b**) Vacuolization in but not outside clumps. Cells were starved in the presence of cAMP for 8 h, further incubated for 16 h with c-di-GMP, and examined by confocal microscopy. Isolated cells close to the glass substrate staid non-vacuolated, whereas cells in the enveloped clump vacuolized and acquired pericellular encasings. Extracted from a Z stack, three slices 10 *μ*m apart. (**c**) Periclump envelopes included cellulose. DH1 (*upper*) or mutant cellulose-less DH1.DcsA- cells (*lower*) were starved in the presence of cAMP for 8 h, then further incubated for 40 h in SB and labeled with calcofluor. The DH1.DcsA- clump was negative for calcofluor fluorescence in spite of deliberate overexposure, confirming that the parental DH1 periclump envelope included cellulose. In this and further experiments, at least five clumps were examined per group. (**d**) Calcofluor staining of periclump envelopes and pericellular encasings. Cells were starved in the presence of cAMP for 8 h, further incubated for 16 h in SB in the absence (upper) or in the presence of c-di-GMP (lower), stained with calcofluor, and examined by confocal microscopy. Slices were extracted from confocal videos. In the absence of c-di-GMP, cells clumped without vacuolization and showed a calcofluor-positive periclump envelope. In the presence of c-di-GMP, cells clumped and vacuolized and showed calcofluor-positive periclump envelopes and pericellular encasings. Scale bars, 10 *μ*m

To induce cell death, following a standard protocol *Dictyostelium* cells were subjected to starvation and cAMP as an initial signal, then to the inducers DIF-1 or c-di-GMP as a second signal. Upon initial signaling by starvation and cAMP, some cells either remained isolated or formed clumps (Figure 1a, left column), recapitulating in part previous results.^4, 8, 9, 10, 11, 12^ These clumps appeared at the end of an 8- h period in the presence of cAMP, became more compact during subsequent incubation without cAMP and then often showed bulges (Figures 1c and d). These bulges were suggestive of morphogenetic initiation,^17, 18^ without, however, evolving into fruiting bodies or macrocysts. Each cAMP-induced clump was surrounded by a calcofluor-positive envelope (Figures 1c and d). This was, however, not observed for DcsA- cells mutated for the cellulose synthase gene (Figure 1c), showing that periclump envelopes included cellulose material. Although both starvation and exogenous cAMP were required for clump formation, for simplicity we shall refer below to cAMP-induced clumps.

**Figure 2:**
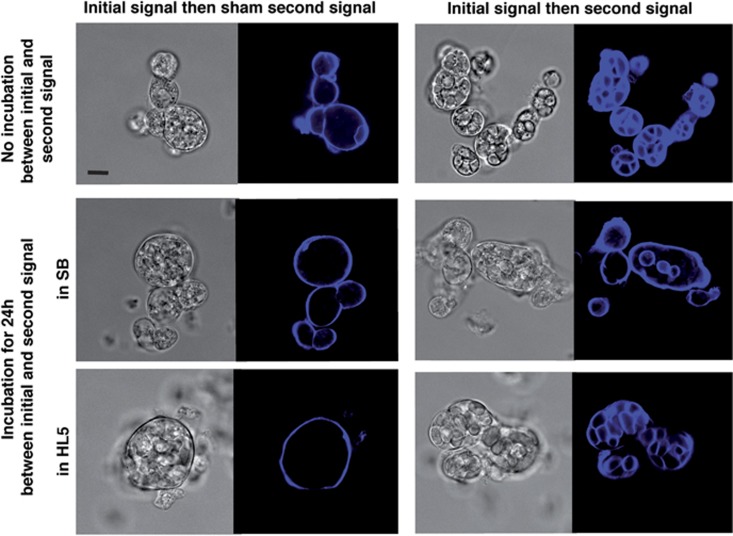
Cells in cAMP-induced clumps showed sustained priming. For these relatively long-term experiments, DH1.DmtA- cells were used, which did not synthesize endogenous DIF-1 and thus did not show ‘spontaneous' vacuolization. Using these cells required as second signal inducers, a mixture of c-di-GMP at the usual concentration of 10 *μ*M and of DIF-1 at the low concentration of 10 nM.^11^ The priming state, as revealed by vacuolization and cellulose encasings only upon addition of inducers (compare left and right panels), persisted even after a further incubation of 24 h in SB (middle row) or HL5 (bottom row) before adding these inducers. Scale bar, 10 *μ*m

In cAMP-induced clumps, second signaling by DIF-1 and/or c-di-GMP led to two major alterations, namely cell vacuolization (Figure 1a) and pericellular cellulose encasings (Figure 1d). DIF-1 induced more clump dissociation than c-di-GMP (Figure 1a). Thus, c-di-GMP preserved spatial segregation between vacuolizing and non-vacuolizing cells, which led us to use it as a second signal in most subsequent experiments. Again, cells subjected to an initial signal and to second signal c-di-GMP vacuolized and acquired a cellulose encasing within clumps, but usually not outside clumps (Figure 1b and see below). cAMP-induced clumps in which c-di-GMP induced vacuolization were observed not only with DH1 cells, but also with AX2 cells of slightly different derivation (http://dictybase.org/strain_history.htm), showing that these results were not restricted to the DH1 background. They were also observed with DH1.DmtA- mutant cells (Figure 2) unable to synthesize DIF-1,^11, 19, 20^ showing that initial signal-induced clumps and priming did not require endogenous DIF-1.

After initial signaling, how long would the primed state persist? DH1.DmtA- cells were primed by starvation and cAMP for 8 h followed by 16 h of starvation. Then second signal inducers were added either immediately (Figure 2, upper row) or after a further 24- h incubation in SB (starvation buffer) or HL5 (rich medium) (Figure 2, middle and lower rows). In all cases, the second signal inducers led to vacuolization and pericellular cellulose encasings, showing that priming persisted even in rich medium for at least 24 h. Cells in cAMP-induced clumps were thus stably primed to vacuolize and acquire cellulose encasings.

Altogether, the initial signal starvation plus cAMP led to the coexistence of isolated cells and clumped cells. The latter were primed to the vacuolization-inducing effect of second signals. In practice, when all cells in the very same preparation, whether within or outside cAMP-induced clumps, were subjected to the same exogenous second signal c-di-GMP, only cells within clumps vacuolized. This made it easier in further studies to distinguish primed from unprimed cells.

### Cells in cAMP-induced clumps showed early and irreversible inhibition of rRNA synthesis

**Figure 3:**
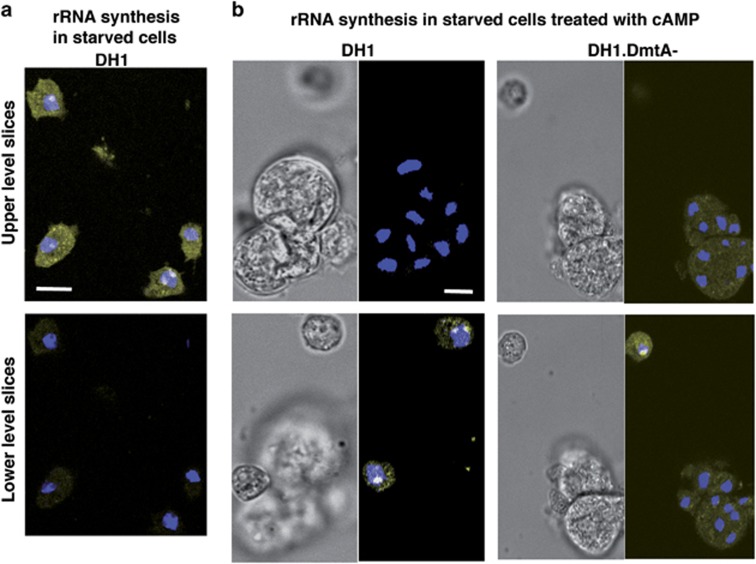
rRNA synthesis in non-clumped cells and its inhibition in cells in cAMP-induced clumps. (**a**) rRNA synthesis in starved cells in the absence of cAMP. In all, 24 h-starved DH1 cells were incubated with EU then fixed, permeabilized, treated with the Alexafluor 488 Click-It reagent, counterstained with DAPI and examined by confocal microscopy. DAPI (pseudocolor blue) and Alexafluor 488 (pseudocolor yellow) pictures were merged. Nucleoli containing newly synthesized rRNA appeared as white bodies at the edge of blue nuclei. Two slices 1.5 *μ*m apart were extracted from the confocal Z stack of Supplementary Video 1, either at lower (close to substrate) or higher levels. White dots in the upper slice, that is, nucleoli containing neo-synthesized rRNA, corresponded to indentations in the DAPI-labeled nuclei in the lower slice. This pattern was found in 17 separate experiments. (**b**) In a separate experiment, DH1 (left) and DH1.DmtA- cells (right) were starved in the presence of cAMP for 8 h, then without cAMP for a total of 24 h. They were then processed as above. Isolated cells outside clumps, lying on the substrate thus best visible in the lower level slices, showed nucleoli labeled through RNA synthesis. Cells in clumps, best visible in the upper level slices, showed no such labeled nucleoli. Upper and lower slices were 10 and 6 *μ*m apart for DH1 cells and DH1.DmtA- cells, respectively. For DH1 cells, slices were extracted from Supplementary Video 2. This pattern was found for each of a total of about 50 examined clumps in 14 (for DH1) and 3 (for DH1.DmtA-) separate experiments. In this and further experiments, nucleolus negativity of a clump usually means that each confocally visible cell in a clump showed no stained nucleolus. Scale bars, 10 *μ*m

Click chemistry to check the biosynthetic incorporation of the uridine analog 5-ethynyluridine (EU) into newly transcribed RNA^21^ was adapted to *Dictyostelium* cells. After 24 h of starvation, incubation for 2 h in the presence of EU, paraformaldehyde (PFA) fixation, permeabilization with triton, staining with AlexaFluor-azide and counterstaining with DAPI, cells showed EU-labeled bodies as yellow-white spots in DAPI-labeled nuclei (Figure 3a and Supplementary Video 1). These bodies were most likely nucleoli, in agreement with the known large proportion of rRNA among newly synthesized RNAs, the localization of this newly synthesized rRNA in nucleoli and the size, place and number of nucleoli in *Dictyostelium* cells.^22, 23, 24, 25, 26^ Each cell showed one nucleolus with one or two lobes, or two nucleoli, at the periphery of its DAPI-labeled nucleus. Each EU-labeled nucleolus often corresponded to a notch at the edge of the DAPI-stained zone in the nucleus (Figure 3a and Supplementary Video 1).

After starvation for 24 h in SB, there was detectable labeling upon incubation for 1 h with 2 mM EU, in HL5 as well as in SB. That detectable rRNA synthesis could take place in SB only, even after 24 h in SB, suggested that rRNA synthesis was a priority even in a starving cell. The nucleoli seemed better defined, with sharper edges, when labeling was in HL5 rather than in SB. Incubation with 4 mM EU in HL5 for 24 h led to no obvious toxicity as judged by cell morphology (data not shown). We used as routine labeling conditions 3 mM EU in HL5 for 2 h in most subsequent experiments.

We then checked RNA synthesis upon signaling for cell death. As shown above, initial signal starvation plus cAMP led to clumps. Clump-containing preparations were incubated with EU. Although isolated cells outside clumps showed EU-labeled nucleoli, cells within clumps showed no such labeling (Figure 3b). This was the case for DH1 cells (Figure 3b, left and Supplementary Video 2) and also for DH1.DmtA- cells (Figure 3b, right) ruling out a role for endogenous DIF-1 in this inhibition of RNA synthesis. Cells outside clumps, which had also been subjected to starvation plus cAMP, did not show this inhibition. Thus, within the same preparations the induction and detection methods used did not interfere with the ability of EU Click-It to reveal EU labeling of nucleoli. Also, the clumps themselves and their cellulose envelopes were unlikely to prevent access to reagents, as all preparations were PFA-fixed and triton-permeabilized, and as cAMP-induced clumps of HMX44A cells forming only incomplete cellulose envelopes also showed inhibition of RNA synthesis (data not shown). From another point of view, we shall consider below that clumping caused RNA synthesis inhibition, although the converse interpretation, that inhibition of RNA synthesis caused cells to clump, was not formally excluded. Altogether, initial signal starvation and cAMP led in nucleoli of clumped cells, but not of isolated cells, to inhibition of EU incorporation, likely reflecting inhibition of rRNA synthesis.

**Figure 4:**
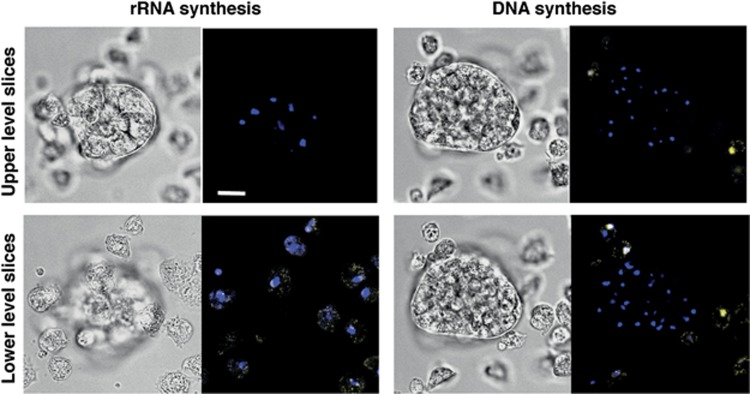
Irreversible inhibition of rRNA and DNA synthesis in cAMP-induced clumps after a further 72- h incubation in rich medium. DH1 cells were starved in the presence of cAMP for 8 h, then without cAMP for 16 h. The resulting clumps were filter-enriched (to avoid subsequent overgrowth in rich medium of too many isolated cells) and incubated for 72 h in rich HL5 medium. DH1 cells incubated with HL5 after the initial signal showed much less of the background vacuolization seen in SB because of some DIF-1 endogenous production by these cells. Similar results were obtained with DH1.DmtA- cells that did not produce DIF-1. EU incorporation assessed rRNA synthesis (left) and EdU incorporation assessed DNA synthesis (right). Isolated cells outside clumps, lying on the substrate thus best visible in the lower level slices, showed labeled nucleoli (left) or nuclei (right). Cells in clumps showed no such labeling. Upper and lower level slices were 10 and 5* μ*m apart for the left and right panels, respectively. Similar results were obtained in two independent experiments. Scale bar, 10 *μ*m

Is inhibition of RNA synthesis irreversible within the duration of these experiments? We usually checked the absence of EU labeling in clumped cells at 16 h after the 8-h cAMP incubation period (Figure 3b). To assess whether this absence was irreversible, we incubated such cells for a further 72 h in rich HL5 medium. Cells in clumps did not regain the capability to incorporate EU (Figure 4, left). Thus, cells in clumps, which at 16 h post-cAMP could not be labeled by EU in contrast to cells outside clumps, were still not able to be labeled following a further incubation of 72 h in HL5. This showed that in clumped cells the rRNA synthesis machinery was irreversibly inhibited at 16 h post-cAMP, ie at the time of addition of the second signal or of HL5.

Not only RNA, but, perhaps as a consequence, also DNA synthesis was irreversibly inhibited in clumped cells. This was shown by checking EdU incorporation into replicating DNA.^27, 28, 29^ After 72 h in HL5, DNA synthesis occurred in isolated cells but not in clumps (Figure 4, right), showing that cells outside clumps cycled, whereas cells within clumps were unable to do so. Thus, cells in clumps were also irreversibly unable to synthesize DNA. Altogether, cells in cAMP-induced clumps were primed to respond to second signals by vacuolizing and at the same time seemed already irreversibly dysfunctional as to both RNA and DNA synthesis.

### Cells in cAMP-induced clumps showed depletion of rRNA stores

**Figure 5:**
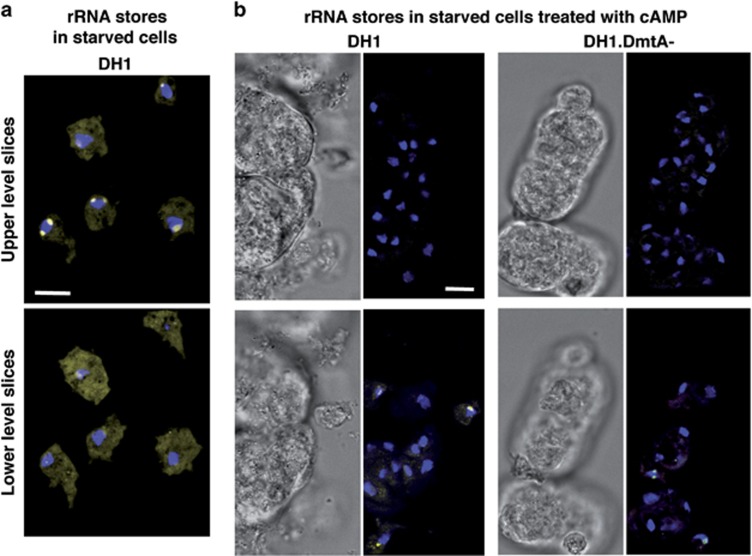
rRNA stores in non-clumped cells and their depletion in cells in cAMP-induced clumps. (**a**) rRNA stores in starved cells in the absence of cAMP. In all, 24 h-starved DH1 cells were fixed, permeabilized, stained with SytoRNAselect and counterstained with DAPI. DAPI (pseudocolor blue) and SytoRNAselect (pseudocolor yellow) pictures were merged. Nucleoli containing rRNA stores appeared as white bodies at the edge of blue nuclei. Lower (close to substrate) and upper slices, 2.5* μ*m apart, were extracted from Supplementary Video 3. In a total of 10 such experiments, each of about 50 examined cells showed a labeled nucleolus. (**b**) In a separate experiment, DH1 (left) and DH1.DmtA- cells (right) were starved in the presence of cAMP for 8 h, then without cAMP for a total of 24 h. They were then processed as above. Isolated cells outside clumps, lying on the substrate thus best visible in the lower level slices, showed nucleoli labeled through their rRNA stores. Cells in clumps, best visible in the upper level slices, showed no such labeled nucleoli. Upper and lower slices were 9 and 10* μ*m apart for DH1 cells and DH1.DmtA- cells, respectively. For DH1 cells, slices were extracted from Supplementary Video 4. The same results were obtained for about 50 examined clumps in a total of 7 (for DH1) and 4 (for DH1.DmtA-) separate experiments. Scale bars, 10 *μ*m

We wished to confirm independently the inhibition of rRNA synthesis by checking remaining rRNA stores rather than ongoing production of rRNA. We treated *Dictyostelium* cells with SytoRNAselect, known to stain preferentially nucleolar rRNA stores in animal cells (Molecular Probes Handbook). In preliminary experiments, *Dictyostelium* cells were starved for 24 h in SB, then fixed and permeabilized as above, stained with SytoRNAselect 500 nM in SB for 2 h and counterstained with DAPI. This resulted in clearcut labeling of nucleoli (Figure 5a and Supplementary Video 3), which also showed that starvation for 24 h did not lead by itself to massive degradation of rRNA.

We then checked rRNA stores in cAMP-induced clumps. Clump-containing preparations were incubated with SytoRNAselect. As for EU incorporation above, isolated cells outside clumps showed labeled nucleoli, cells within clumps showed no such labeling, and this was the case for DH1 (Figure 5b, left and Supplementary Video 4), as well as for DH1.DmtA- cells (Figure 5b, right). Cells outside clumps, which had also been subjected to starvation plus cAMP, still showed SytoRNAselect staining. These and previous results strongly suggested that, while in cells outside cAMP-induced clumps rRNA nucleolus stores were continuously fed by ongoing rRNA synthesis, in cells within clumps such stores were depleted because of inhibition of rRNA synthesis and exhaustion of previous rRNA stores. They showed, moreover that rRNA stores in cells within cAMP-induced clumps persisted for <24 h under these conditions, resulting in their absence at the time of second signaling.

### Cells in cAMP-induced clumps showed ultrastructural disorganization of nucleoli

**Figure 6:**
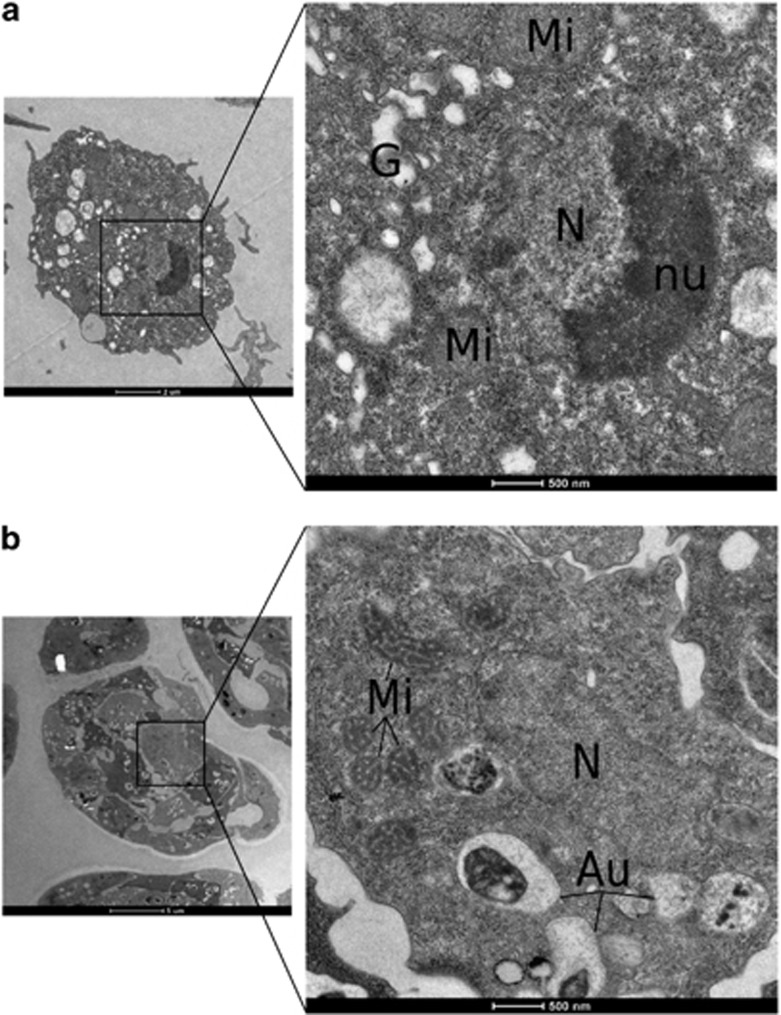
Nucleoli prominent in vegetative cells were disorganized in cells in cAMP-induced clumps. (**a**) Ultrastructural morphology of a representative vegetative cell in rich medium. Scale bar, 2* μ*m. The higher magnification shows the nucleus with its more electron-dense nucleolus. This was the case for each of 10 examined vegetative cells. G, Golgi; Mi, mitochondria; N, nucleus; nu, nucleolus. Scale bar, 0.5* μ*m. (**b**) Ultrastructural morphology of a representative cAMP-induced cell clump. Scale bar, 5 *μ*m. The higher magnification shows the nucleus of one cell in the clump, with no identifiable nucleolus. This was the case for every cell in each of five examined clumps. Au, probable autophagosomes. Scale bar, 0.5 *μ*m

In view of the known relationship between rRNA and structure of nucleoli (see Discussion section), we wondered whether nucleoli would be altered in *Dictyostelium* clumped cells showing inhibition of rRNA synthesis. DH1.DmtA- cells, either vegetative and washed in SB, or cAMP-induced 16-h clumps enriched by filtration, were processed for electron microscopy. Vegetative cells showed prominent electron-dense nucleoli (Figure 6a). Cells in cAMP-induced clumps showed barely visible nucleoli (Figure 6b) and no other gross morphological lesions except for the autophagosomes expected in these starving cells. Altogether, and very likely in line with rRNA synthesis inhibition, cAMP-induced clumped cells showed disorganization of their nucleoli.

## Discussion

**Figure 7:**
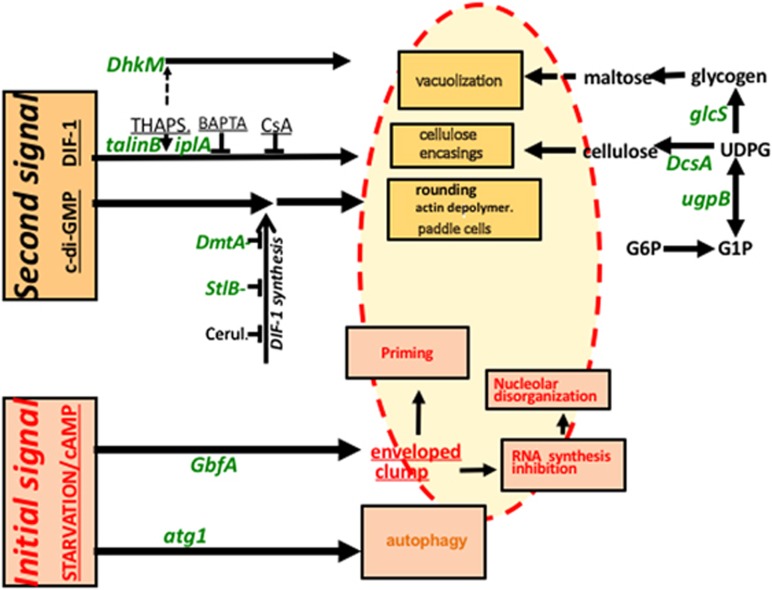
A schematic representation of *Dictyostelium* cell death. As detailed in the present article, most subcellular cell death events took place in enveloped clumps (here shown as a yellow area limited by a red dotted line). Such clumps were induced by the initial signal starvation/cAMP. In clumps, further events, in particular those indicated in pink boxes with red letters, ultimately led both to priming to second signal and to commitment to die. The second signal DIF-1 or c-di-GMP induced cell death hallmarks, such as vacuolization and cellulose encasings, indicated in beige boxes with black letters. Random insertional mutagenesis and targeted mutagenesis have identified a number of genes, here shown in green letters, encoding molecules required for inducing this cell death or its hallmarks.^12^ These genes in turn helped defined pathways, such as a polysaccharide pathway (right), and the pathways triggered by the initial and the second signals mentioned above (left)

Our current understanding of cell death events in *Dictyostelium*, including the expanded consequences of initial signaling described in this report, is schematized in Figure 7. The initial signal (starvation and cAMP) induced clumps where cells were not only primed for second-signaled vacuolization, but also, most importantly, were already severely dysfunctional. Indeed, upon initial signaling, nucleoli were disorganized and rRNA and DNA synthesis were irreversibly inhibited. In the absence of second signal, these clumped and dysfunctional cells did not show immediate loss of membrane integrity (data not shown). Upon second signaling, these very same cells acquired cell death hallmarks such as vacuoles and cellulose encasings. They must end up dying in the process, but we are unable to decide when, in particular because of a lack of unambiguous definition of the moment of cell death. For this reason, we do not know whether the second signal only induces cell death hallmarks or also contributes to death, through these hallmarks or otherwise.

We found clearcut differences between cells within and outside cAMP-induced clumps in terms of rRNA synthesis. The proportion of cells within and outside clumps (or in similar developmental situations) may therefore affect the results of some transcriptomic analyses. Still, in spite of some possible averaging and of markedly different experimental situations, in line with the present results such analyses showed downregulation of transcription early upon development for genes related to ribosome biogenesis.^30^ Similarly, the rate of rRNA synthesis during *Dictyostelium* development (however, again averaging all cell types) was previously found to be <15% of that of growing cells.^31^ From another point of view, in initial signal-induced clumps there was synthesis of periclump cellulose envelopes and upon second signaling of pericellular cellulose shells, implying the presence and activity of the inducible *Dictyostelium* cellulose synthase,^3^ indicating that in clumped cells RNA synthesis was not completely inhibited or that these events were post-transcriptionally controlled.

In the present report, three different approaches, namely studies of rRNA synthesis by EU Click It tests, of rRNA storage by SytoRNAselect staining and of nucleolar morphology by electron microscopy, showed in cAMP-induced clumped cells consistent inhibition of rRNA synthesis, depletion of rRNA stores and nucleolar disorganization. These events seemed causally related. The initial signal led to clumping and inhibition of rRNA synthesis, which led to depletion of rRNA stores and led to nucleolar disorganization as discussed more in detail below. rRNA-related changes in the structure of nucleoli likely reflected their status as rRNA-dependent ‘droplet organelles'.^32, 33^ In experiments not shown, we investigated whether, for priming, inhibition of RNA synthesis (and subsequent rRNA store depletion and nucleolus disorganization) could replace the initial signal. Starving vegetative cells were subjected to actinomycin D. In some experiments, clump supernate was also added. Even then, subsequent addition of DIF-1 and/or c-di-GMP led to no vacuolization, showing that RNA synthesis inhibition, even together with starvation and clump supernate, was not sufficient for priming. We do not know which soluble substances acting at short distance and/or cell contacts may (also) be required for priming in cAMP-induced clumps.

To our knowledge, there is only one report of alterations of nucleoli in *Dictyostelium* cell death, induced by exocytotic vesicles purified from starved *Dictyostelium* cells.^34^ A relationship between rRNA synthesis, assembly of ribosomes and nucleoli was observed in *Dictyostelium* cells^22, 23, 35^ where nucleolar subcompartments could be evidenced.^26^ Changes in the structure of nucleoli were described upon development^24, 25, 36^ and upon actinomycin D-induced inhibition of RNA synthesis.^22, 24, 35^

In animal cells, synthesis of rRNA and pre-assembly of ribosomes were shown to take place in nucleoli (reviewed in Pederson^37^), and inhibition of rRNA synthesis by actinomycin D^38, 39, 40, 41, 42, 43^ or CX-5461 (refs 44, 45, 46, 47) led to nucleolar disorganization and, interestingly, eventually apoptosis.

More specifically in mammalian cells, the nucleolus was identified as a sensor of stress and an executor of resulting lesions.^48, 49, 50, 51, 52^ Stress activated JNK, leading to inactivation of the TIF-1A transcription factor,^53^ which normally regulated the activity of RNA polymerase 1 toward transcribing rRNA. Inactivation of TIF-1A thus led to inhibition of rRNA synthesis and consequently to nucleolar disorganization. In turn, nucleolar disorganization resulted in loss of sequestration in nucleoli of ribosomal proteins, which were released in the cytosol, formed complexes with MDM2 and thus prevented it from ubiquitinylating p53. The latter could then induce apoptosis.^54, 55, 56, 57, 58, 59, 60^ Interestingly, nucleolar stress and its consequences could also occur in cells lacking MDM2 or p53.^61^ Of note, JNK could be activated not only by stress, but also by BMP signaling, which was required for interdigital^62, 63^ and other instances of developmental cell death (Pachori *et al.*^64^ and references therein).

RNA synthesis inhibition and nucleolar alterations were indeed noted in early apoptosis papers. For instance, in rat thymocytes treated with glucocorticoids, early in apoptosis ‘nucleolar constituents underwent segregation and dispersal' ^65^ and there was <5% of the rate of incorporation of uridine seen in control cells.^66^ While in *Dictyostelium* cell death nucleolar disorganization occurred in the absence of early DNA fragmentation^9^ and of caspases,^67, 68^ nucleolar changes upon apoptosis have been attributed to cleavage of DNA by endonucleases^65^ or to caspase-dependent proteolysis.^69^ Altogether, diverse instances of animal cell death including developmental apoptosis included nucleolar disorganization.

The results reported here on early nucleolar disorganization in non-apoptotic developmental cell death in a protist and previous similar observations in animal apoptotic cell death suggested the following hypothesis. In some cases of eukaryotic cell death a first, early stage would encompass nucleolar disorganization and priming. This stage may show a degree of conservation between some types of cell death throughout eukaryotes, and may thus be in line with ubiquity of cell death. A second stage, triggered by an exogenous second signal in *Dictyostelium* but also perhaps in other eukaryotes could follow less conserved mechanisms, show aspects dependent on local mechanical constraints and expression of, for example, given proteases,^70^ and thus lead to various morphological types of cell death. Second signal-induced pathways and resulting morphological types could thus be specific to organism. This hypothesis would reconcile, in at least some instances of eukaryotic developmental cell death, conservation of some initial stages of cell death with polymorphism at later stages.

## Materials and methods

### Induction of cell death in monolayers

*Dictyostelium* cells were DH1 (initially obtained from RH Kessin, Columbia University, New York, NY, USA) unless stated otherwise. DH1 cells mutated for the cellulose synthase DcsA (DH1.DcsA-) (this report) or the methyltransferase DmtA (DH1.DmtA-)^11^ were also used. Vegetative cells were collected in log phase from cells grown in Falcon flasks in HL5 culture medium, washed twice in phophate-buffered saline (Sörensen buffer, SB), resuspended in SB containing 3 mM cAMP (Sigma Aldrich, St Louis, MO, USA A6885), and distributed at 3–6 x10e5 cells in 1 ml of SB plus cAMP per chamber of 2-chamber-LabTek slides (155380, Nalge Nunc, Penfield, NY, USA). Cells were incubated for 8 h at 22 °C, then the liquid was carefully removed by aspiration followed by one wash with 1 ml of SB per chamber, which was replaced with 1 ml of SB containing either no inducer when only initial signal effects were investigated, or, as second signals, 100 nM DIF-1 (DN1000, Affiniti Research Products, Exeter, UK) or more often here and 10 *μ*M c-di-GMP sodium salt (C 057-01; Biolog, Bremen, Germany) or a mixture of 10 nM DIF-1 and 10 mM c-di-GMP.^11^ Further incubation at 22 °C was usually for 16 h. The cells were thus kept under starvation for a total of 24 h, namely 8 h with cAMP plus 16 h without cAMP in SB only or in the presence of inducers. In some case, detailed in the main text, incubation proceeded for longer times. Cells were directly examined by phase contrast or labeled as indicated below.

### Imaging RNA synthesis

We used the Click-It RNA Imaging kit (C10329, Molecular Probes Invitrogen, Eugene, OR, USA), following the provider's protocol with some modifications. Per Labtek chamber, cells starved for 24 h with or without cAMP for 8 h were incubated with EU (final 3 mM for 2 h, unless stated otherwise), then fixed with PFA (final 1%) removed after 15 min, permeabilized with triton (final 0.5% for 20 min), then treated with the Alexafluor 488 Click-It reagent, rinsed once with SB and counterstained with DAPI 0.1 *μ*g/ml in SB for 10 min. No wash was required.

### Imaging RNA stores

We used the SYTO RNASelect Green Fluorescent Cell Stain (S32703, Molecular Probes Invitrogen), following the provider's protocol with some modifications. Per Labtek chamber, cells starved for 24 h including or not cAMP for 8 h were fixed with PFA and permeabilized with triton as above. Then triton was discarded, cells were incubated with SytoRNAselect (200 nM in SB, for 2 h, protected from light), rinsed once with SB and counterstained with DAPI as above.

### Clump enrichment by filtration

**C**lump formation (SB plus cAMP for 8 h, then SB only for the following 16 h) was obtained by incubating 10^7^ cells/10 ml SB in Petri dishes not treated for tissue culture (Greiner Bio-One, Kremsmunster, Austria, ref 633185). The resulting 10 ml suspension containing cells and cell clumps was then filtered on a pluriStrainer 20 *μ*m (pluriSelect Life Science, Leipzig, Germany) filter prewashed with 2 ml SB. The filter was then inverted, and the retained clumps were eluted with 10 ml SB or HL5. The suspension was either distributed in LabTeks (1 ml per chamber) for long-term reversibility experiments or processed for electron microscopy.

### Imaging DNA synthesis

The Click-It Edu Plus Fluor 488 kit from Molecular Probes/Life Technologies (Eugene, OR, USA) (ref C10637) was used according to the provider's protocol, slightly modified as follows. Suspensions of filtration-enriched clumps were distributed in LabTek chambers and were left to sediment for at least 1 h. In each LabTek chamber, the supernate was replaced by 1 ml HL5 containing EdU Plus 500 *μ*M final. This was in line with the relatively high concentrations of BUdR previously used in *Dictyostelium*.^71, 72^ Lower concentrations of EdU led to much weaker labeling. Then incubation proceeded for 72 h. In each LabTek chamber, a volume of 400 *μ*l of PFA 4% in SB was then added. After 15 min, PFA was removed and replaced by 1 ml of Triton 0.5% in SB for 20 min, replaced by 1 ml of BSA 2% in SB for 15 min, replaced by 0.5 ml of freshly prepared Click IT mix for 30 min protected from light. After one wash in SB, DAPI 0.1 *μ*g final in 1 ml of SB was added. Examination by fluorescence microscopy could take place either immediately or better after one day (which led to less background).

### Imaging cellulose

Cellulose staining was with calcofluor (as fluorescent brightener 28, Sigma F3543). This powder was dissolved in water (1% weight/volume). This stock solution was further diluted 1 : 10 in water, of which a volume of 10 *μ*l was added to 1 ml of SB per LabTek chamber (thus final concentration 1/1000). No wash was required and observation was after 5–10 min.

### Microscopy

Cells were examined and photographed through the glass bottom of LabTek chambers, either by phase contrast microscopy (Axiovert 200 M Carl Zeiss, Oberkochen, Germany; x100 oil immersion) or by confocal microscopy (Leica SP5, Wetzlar, Germany; x63 oil immersion). Fluorochromes were Alexafluor 488 (excitation 488 nm, emission 525 nm) and SYTO RNAselect (excitation 490 nm, emission 530 nm). Confocal Z-stacks were usually started from the glass substrate upward, thus taking isolated cells first and then clumped cells. Control reverse Z-stacks gave the same results, showing that there were no position/fading misleading effects. Confocal videos and other images were processed with Fiji (Image J, NIH, Bethesda, MD, USA) and Graphic Converter. DAPI (pseudocolor blue) and Alexafluor 488 or SYTO RNAselect (pseudocolor yellow) pictures were merged. Nucleoli containing newly synthesized rRNA or rRNA stores appeared as white bodies at the edge of blue nuclei. Figures were assembled using Illustrator.

### Transmission electron microscopy

Cells or cell clumps were prefixed by adding an equal volume of fixative (2% glutaraldehyde in Hepes buffer 200 mM, pH 7.2) to the medium. After 20 min, the medium was replaced by 1% glutaraldehyde in Hepes for at least 1 h at 4 °C. Cells were then washed in Hepes, concentrated in 2% agarose (LMP Agarose, Sigma A9414), washed again in Hepes and postfixed in 1% osmium tetroxide (Electron Microscopy Sciences, Hatfield, PA, USA 19150) for 1 h at 4 °C. Samples were washed again in distilled water, treated with 1% uranyl acetate (EMS 22400) for 1 h at 4 °C in the dark, dehydrated in a graded series of acetone and embedded in Epon resin (EMS). Ultrathin sections (60–90 nm) were cut, stained with uranyl acetate and lead citrate and were analyzed using a Tecnai 200 KV (operated at 120 KV) electron microscope (FEI Tecnai, Hillsboro, OR, USA).

### Preparation of DH1.DcsA- cells by targeted mutagenesis

Targeted mutagenesis of the cellulose synthase *DcsA* gene^3^ in DH1 cells was by homologous recombination, by deletion from nt677 to nt2576 in the *DcsA* gene. The targeting vector was constructed as follows. A *DcsA* DNA fragment made of a 5′ arm (nt2-nt677) and a 3′ arm (nt2576-nt3099) PCR-amplified from gDNA and ligated by PCR, was cloned into the pGEM-T Easy Vector (Promega, Madison, WI, USA). A bsR cassette was inserted in the *Bam*HI site between the two *DcsA* arms. These constructs were validated by digestions and sequencing. DH1 *Dictyostelium* cells were transfected by electroporation (1 kV; 3 *μ*F using a Bio-Rad gene pulser, Hercules, CA, USA) with the *DcsA* construct after digestion by *Msc*I and *Hha*I. Cells were selected for resistance to 10 *μ*g/ml blasticidin, and then cloned by limiting dilution. Homologous recombinaison was checked by PCR and verified by Southern blot. STOP codons at the 5′ end of the bsR cassette should ensure a maximum length of 225 aa for the *DcsA* mutant protein. Primers were: *DcsA*-Arm5′- S: 5′-GGATAGAAATGAAGGGGGTGATTTCCC-3′ *DcsA*-Arm5′-AS: 5′-GGATCCGTTTCAGAATCTTCTTTGGCGAC-3′; *DcsA*-Arm3′-S: 5′-GGATCCGGGTAGAAGCTACTGATCTTTGGAGAGC-3′; *DcsA*-Arm3′-AS: 5′-GTGAGCATGGTATGAAGAAGCATATGGCCATTG-3′.
